# Critical role of sigma-1 receptors in central neuropathic pain-related behaviours after mild spinal cord injury in mice

**DOI:** 10.1038/s41598-018-22217-9

**Published:** 2018-03-01

**Authors:** Sílvia Castany, Georgia Gris, José Miguel Vela, Enrique Verdú, Pere Boadas-Vaello

**Affiliations:** 1 Research Group of Clinical Anatomy, Embryology and Neuroscience (NEOMA), Department of Medical Sciences, Universitat de Girona (UdG), Girona, Spain; 2 ESTEVE, Drug Discovery and Preclinical Development, Parc Científic de Barcelona, Barcelona, Catalonia Spain

## Abstract

Sigma-1 receptor (σ_1_R) knockout (KO) CD1 mice, generated by homologous recombination, and separate pharmacological studies in wild type (WT) mice were done to investigate the role of this receptor in the development of pain-related behaviours (thermal hyperalgesia and mechanical allodynia) in mice after spinal cord contusion injury (SCI) – a model of central neuropathic pain. The modulatory effect of σ_1_R KO on extracellular mediators and signalling pathways in the spinal cord was also investigated. In particular, changes in the expression of inflammatory cytokines (tumour necrosis factor TNF-α, interleukin IL-1β) and both the expression and activation (phosphorylation) of the *N*-methyl-D-aspartate receptor subunit 2B (NR2B-NMDA) and extracellular signal-regulated kinases (ERK1/2) were analysed. Compared with WT mice, both mechanical and thermal hypersensitivity were attenuated in σ_1_R KO mice following SCI. Accordingly, treatment of WT mice with the σ_1_R antagonist MR309 (previously developed as E-52862; S1RA) after SCI exerted antinociceptive effects (i.e. reduced mechanical allodynia and thermal hyperalgesia). Attenuated nociceptive responses in σ_1_R KO were accompanied by reduced expression of TNF- α and IL-1β as well as decreased activation/phosphorylation of NR2B-NMDA receptors and ERK1/2. These findings suggest that σ_1_R may modulate central neuropathic pain and point to regulation of sensitization-related phenomena as a possible mechanism.

## Introduction

Spinal cord injury (SCI) may trigger central neuropathic pain, which is defined as the pain caused by a lesion or disease of the central somatosensory nervous system^1^. Prevalence rates for SCI-related neuropathic pain are over 50% and such pain states are in general refractory to treatment and have a poor prognosis^2^. Central neuropathic pain is a serious health condition, with considerable impact on patients’ quality of life and high individual and societal economic burden^3^. It is thus imperative to focus research efforts on identifying new therapeutic approaches.

Among possible new pharmacological strategies, we assessed here the modulation of the sigma-1 receptor (σ_1_R), a neuromodulatory, ligand-regulated membrane protein chaperone that exerts its function through multiprotein complex assembly^4–7^. σ_1_R is expressed in important pain control areas in the central nervous system^8–10^, where it regulates neuronal plasticity and activity-dependent sensitization^11–14^. Remarkably, findings at the electrophysiological (spinal wind-up recordings), neurochemical (spinal release of neurotransmitters) and molecular (NMDAR function) level support a role for σ_1_R antagonists in inhibiting the amplified response of spinal cord neurons to sustained afferent input^14^. Electrophysiologically, σ_1_R antagonists inhibited the spinal wind-up amplification phenomenon when trains of nociceptive stimuli were applied to isolated spinal cords^13,15^, and spinal wind-up amplification was reduced in spinal cords from σ_1_R KO compared to WT mice^12^. Accordingly, in neurochemical studies (i.e., dorsal horn microdialysis), σ_1_R antagonist (MR309; S1RA)–mediated antinociception was shown to promote plastic descending inhibitory pathways (enhanced noradrenaline levels) and stopped down plastic excitatory synaptic strengthening (attenuated glutamate release) in the dorsal horn. Sustained glutamate release in the dorsal horn is known to promote plastic changes leading to central (spinal) sensitization phenomena and, among glutamate receptors, the NMDAR is key for such activity-dependent plasticity underlying pain sensitization/hypersensitivity^16–19^. As shown at the molecular level, the σ_1_R interacts with and is functionally coupled to NMDAR^20–22^ and this interaction accounts for its modulatory effects: σ_1_R antagonists decreased glutamate NMDAR currents, and NMDA current was shown to be reduced in σ_1_R KO respect to WT mice^23^. Interestingly, σ_1_R is reported to play a role in regulating stress-response signalling and ultimately in cell survival^4,7^.

The present study comprised two independent sets of experiments to investigate the role of this chaperone protein in the development of central neuropathic pain-related behaviours after mild spinal cord contusion injury in mice. In the first set of experiments (Part 1), we used homozygous σ_1_R KO (σ_1_R^−/−^) mice generated by homologous recombination^15^, and in the second set of studies (Part 2) we utilized pharmacological blockade.

In previous studies, σ_1_R KO animals have been used to investigate the role of σ_1_R in animal models where pain was primarily induced at the periphery, such as paw capsaicin and formalin injection^24,25^, inflammatory pain models^26,27^, and peripheral neuropathic models, including sciatic constriction injury^12^ and paclitaxel-induced neuropathy^28^. In all these models, pain-related behaviours (e.g., formalin-induced paw licking/biting, capsaicin-induced mechanical allodynia or nerve injury-induced mechanical allodynia) were absent or attenuated in σ_1_R KO mice compared to WT mice. However, the phenotype of σ_1_R KO mice when exposed to a central nervous system injury, and spinal cord contusion in particular, remains unknown. To this end, in the present study WT and σ_1_R KO mice were subjected to spinal cord contusion^29^ and their responses to mechanical and thermal stimulation recorded up to 4 weeks after injury.

As a mechanistic correlate, given the role of σ_1_R in modulating central sensitization phenomena^14,30^, we also investigated phosphorylation in the spinal cord of the glutamate *N*-methyl-D-aspartate (NMDA) receptor and the extracellular signal-regulated kinase (ERK1/2); both of which are reportedly involved in central sensitization^19^ and hypersensitivity in neuropathic pain conditions^31,32^. Moreover, considering the role of pro-inflammatory cytokines in the pathophysiology of neuropathic pain after SCI, the expression of TNF-α and IL-1β^33–35^ was investigated.

Finally, in order to study the role of σ_1_R in the expression of SCI-induced neuropathic pain (i.e., once pain had developed), data from σ_1_R KO mice were complemented with pharmacological data obtained from separate studies in which the σ_1_R antagonist MR309 (S1RA) was administered to SCI WT mice. MR309 is a selective σ_1_R antagonist that has demonstrated efficacy in multiple translationally-driven peripheral neuropathic pain models^36^, but no information is available as it regards to its potential antinociceptive effect in central neuropathic pain models (i.e., spinal cord contusion). Thus, the main objectives of the present work were to study the involvement of σ_1_R in mild SCI-induced central neuropathic pain through genetic (σ_1_R KO mice) and pharmacological (MR309 administration, dose-response study in WT mice) approaches.

## Results

### General observations and mice genotyping

Following a protocol animal welfare supervision based on Morton D.B and Griffiths P.H. guidelines (1985)^37^, changes in coat and skin, vibrissae of nose, nasal secretions, signs of autotomy of hindpaw and/or forepaw, or aggressiveness were not detected neither in WT nor σ_1_R KO mice after SCI at any time of the experimental period. Animals showed no significant weight loss throughout the experiment.

### Locomotor disturbance in σ_1_R KO and WT mice after mild SCI

**Figure 1:**
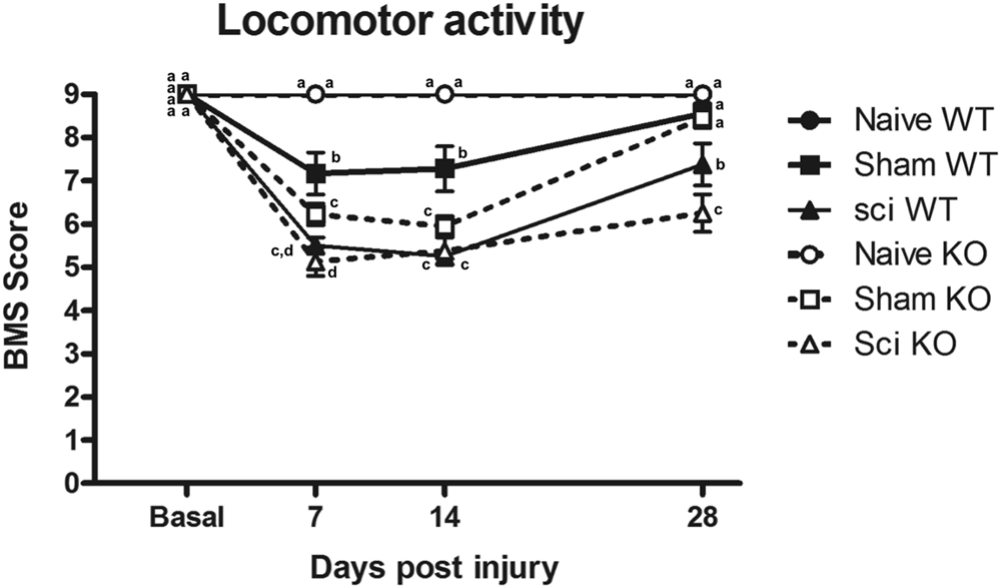
Locomotor recovery assessment using Basso Mouse Scale (BMS) after spinal cord injury (SCI) in wild type (WT) and sigma-1 receptor (σ_1_R) knockout (KO) mice. Each point and vertical line represents the mean ± standard error of the mean (n = 9–12 per group). a–c: groups not sharing a letter are significantly different, p < 0.05. Results reveal mild BMS alteration associated with SCI in both WT and σ_1_R KO mice, referring to altered paw position but not to altered horizontal locomotion.

In preclinical studies, SCI may be classified as mild, moderate or severe according to the motor dysfunction displayed by the injured animals^38,39^, which it is usually evaluated by means of Basso Mouse Scale for locomotion (BMS)^40^, among other available locomotor functional tests. Thus, SCI may be classified as mild (BMS-scores >6), moderate (BMS-scores 4–5) or severe (BMS-scores <4) according to the motor dysfunction following injury. In previous studies, 2g-weight contusion was shown to result in mild locomotor disturbances without paralysis in WT animals^41^. Thus, we first evaluated whether the same contusion procedure produces the same effect in σ_1_R KO mice. To this end, a BMS test^40^ was used to compare locomotor function up to 4 weeks after SCI. Multivariate analysis of variance (MANOVA) revealed significant effects on day (F_(3,48)_ = 180.22, p < 0.001), surgery (F_(2,50)_ = 170.73, p < 0.001) and genotype (F_(1,50)_ = 5.49, p = 0.05) factors and significant interactions for day × surgery (F_(6,96) = _32.803, p < 0.001) and day × genotype (F_(3,48)_ = 2.82, p < 0.05). Moreover, significant group differences were detected by analysis of variance (ANOVA) analysis in BMS scores at 7, 14 and 28 (all p values < 0.001) days post-injury (dpi) (Fig. 1). While at 7 dpi both sham and contusioned experimental groups showed a deficit in coordination compared to naïve groups from both genotypes, at 28 dpi only contusioned WT and σ_1_R KO mice showed significant lower BMS scores with respect to all other (naïve and sham) groups (Fig. 1). No remarkable differences were found when compared WT and σ_1_R KO mice except for slightly higher locomotor impairment transiently seen in sham KO *versus* sham WT mice at 7 and 14 dpi, but not at 28 dpi, and in SCI KO versus SCI WT at 28 dpi. By the last day of evaluation (28 dpi), impairment remained significant in WT and σ_1_R KO mice subjected to SCI (but not in sham groups). According to BMS scale, the rating scores (mean ± SEM) of these groups (WT = 7.4 ± 0.40; σ_1_R KO = 6.25 ± 0.44) denoted an impairment slightly higher in KO mice subjected to SCI, but only altered paw position and no altered horizontal locomotion, indicating no major impairment in coordination and locomotor function, was scored in both genotypes. In summary, values above 6 in the BMS test were obtained in mice of both genotypes subjected to SCI, indicating only mild locomotor dysfunction without major impairments. Overall, neither sham surgeries nor spinal cord contusion resulted in either paralysis or major locomotor dysfunction at 28 dpi, at any experimental group.

### Attenuation of mechanical allodynia and thermal hyperalgesia in σ_1_R KO mice after SCI

σ_1_R is known to be involved in the modulation of neuropathic pain after painful peripheral nerve injury^5,10,42^. In this study we evaluated and compared the response to mechanical and thermal stimulation of σ_1_R KO and WT in the SCI model up to 4 weeks after SCI.

**Figure 2:**
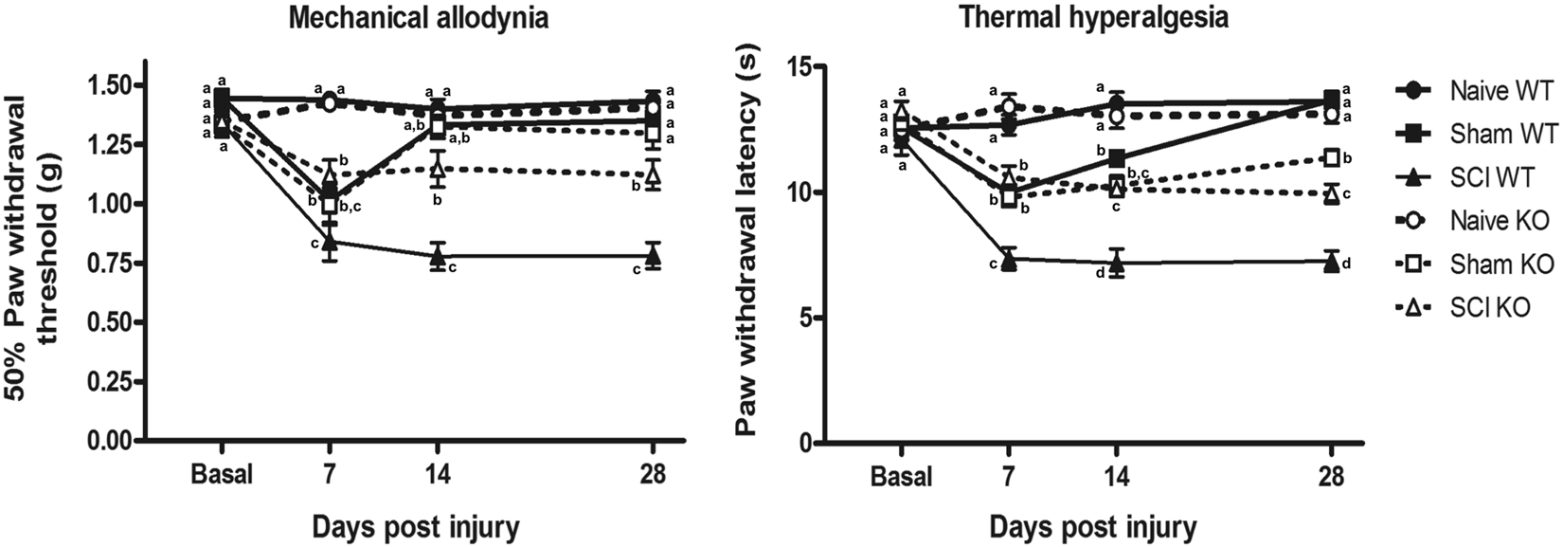
Time course of spinal cord injury (SCI)-induced mechanical allodynia and thermal hyperalgesia in wild type (WT) and sigma-1 receptor (σ1R) knockout (KO) mice. Each point and vertical line represents the mean ± standard error of the mean (n = 9–12 per group). a–c: groups not sharing a letter are significantly different, p < 0.05. (**A**) Mechanical allodynia and (**B**) thermal hyperalgesia was clearly evidenced on all measurement days in SCI WT mice. The hypersensitivity was attenuated in homozygous σ1R KO mice on days 7, 14 and 28 after SCI.

Mechanical allodynia was assessed via measurement of hind paw withdrawal threshold in response to von Frey filament stimulation^43^. The MANOVA analysis indicated significant effects on day (F_(3,48)_ = 22.814, p < 0.001), surgery (F_(2,50)_ = 78.85, p < 0.001) and genotype (F_(1,50)_ = 5.49, p = 0.023) factors and significant interactions for day × surgery (F_(6,96)_ = 13.927, p < 0.001) and day × genotype (F_(3,48)_ = 4.536, p < 0.001). On further ANOVA analysis, significant group differences were found on post-injury days 7, 14 and 28 (all p values < 0.001) (Fig. 2A). Naïve animals from both genotypes did not show mechanical allodynia throughout the experimental period, and no differences in mechanical sensitivity were found when compared naïve mice from both genotypes. Similarly, no differences were found when comparing sham mice of both genotypes. Sham-operated mice showed a significant decrease (p value < 0.05, Duncan test) in mechanical paw withdrawal thresholds at 7 dpi when compared with naïve mice, but mechanical allodynia was markedly attenuated at 14 dpi and was absent at 28 dpi in sham mice from both genotypes. Mechanical allodynia developed following SCI in both σ_1_R KO and WT, but the time course and severity were different when both genotypes were compared. By 7 dpi mechanical allodynia clearly developed in SCI mice (similar to sham mice), but it was attenuated in SCI σ_1_R KO when compared with SCI WT mice. At 14 dpi mechanical allodynia was apparent in SCI (but not in sham-operated) mice and reduced in SCI σ_1_R KO compared with SCI WT mice. Finally, at 28 dpi, mechanical allodynia was markedly reduced in SCI σ_1_R KO compared with SCI WT. Indeed, σ_1_R KO mice subjected to a SCI showed an average 54% reduction in mechanical allodynia at 7, 14, and 28 dpi when compared to WT SCI mice.

Thermal hyperalgesia was assessed by measuring hind paw withdrawal latency in response to thermal stimulation (radiant heat) in the plantar test^44^. The MANOVA analysis indicated significant effects on day (F_(3,48)_ = 28.853, p < 0.001), surgery (F_(2,50)_ = 123.64, p < 0.001) and genotype (F_(1,50)_ = 15.1, p = 0.04) factors and significant interactions for day × surgery (F_(6,96)_ = 21.07, p < 0.001) and day × genotype (F_(3,48)_ = 2.703, p < 0.05). On further ANOVA analysis, significant group differences were found on post-injury days 7, 14 and 28 (all p values < 0.001) (Fig. 2B). Similar to mechanical allodynia, thermal hyperalgesia did not develop throughout the experimental period in naïve animals, and no differences in thermal sensitivity were found when compared naïve mice from both genotypes. A significant decrease in paw withdrawal latency (i.e. thermal hyperalgesia) was found in sham mice at 7 and 14 dpi (p values < 0.05, Duncan test) when compared with naïve mice. At the end of the experimental period (day 28), thermal hyperalgesia was absent in WT subjected to sham surgery, but a slight hyperalgesia still remained in sham σ_1_R KO mice. SCI induced a marked and long- lasting thermal hyperalgesia in WT mice, already remarkable at day 7 (significantly higher than in sham groups; p values < 0.05, Duncan test) and maintained throughout the experimental period. Thermal hyperalgesia was markedly attenuated in SCI σ_1_R KO respect to SCI WT mice at all time points. Indeed, σ_1_R KO mice subjected to SCI showed an average 51% reduction in thermal hyperalgesia at 7, 14, and 28 dpi when compared with WT SCI mice.

Altogether, although baseline perception of sensory mechanical and thermal stimuli was similar in σ_1_R KO and WT mice, as evidenced by indistinguishable mechanical thresholds and thermal latencies for paw withdrawal in naïve mice of both genotypes, mechanical and thermal hypersensitivity induced by a spinal cord contusion were significantly lower (p values < 0.05, Duncan test) in σ_1_R KO animals compared with WT mice.

### Differences on central sensitization-related molecular biomarkers between σ_1_R KO and WT mice after SCI

In order to gain mechanistic insights, the spinal expression and phosphorylation of extracellular signal-regulated kinases (ERK1/2) and NMDA receptor NR2B subunit, which have been reported to be involved in central sensitization in neuropathic pain states^31,32,45,46^, were investigated 28 days after injury.

**Figure 3:**
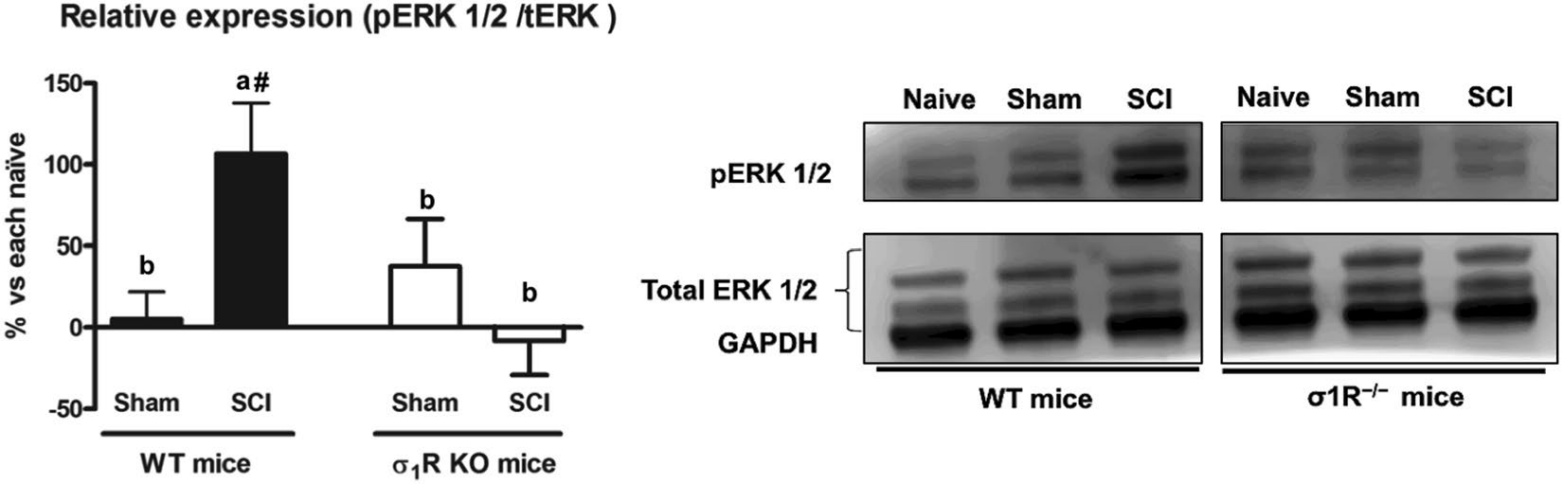
Spinal ERK1/2 phosphorylation (pERK) expression at day 28 after spinal cord injury (SCI) in wild type (WT) and sigma-1 receptor (σ1R) knockout (KO) mice. Quantification and representative immunoblots of total ERK, pERK and glyceraldehyde 3-phosphate dehydrogenase (GAPDH). Protein expressions were normalized to GAPDH and data is presented as a percentage respect to WT naïve or KO naïve mice (mean ± standard error of the mean; n = 5–6). a–b: groups not sharing a letter are significantly different, p < 0.05; ^#^significant differences vs. naïve (p < 0.05). σ1R KO mice subjected to a spinal cord contusion did not show significant upregulation of pERK in contrast to WT SCI mice. Full-length blots are presented in Supplementary Figure S1.

Significant group differences were detected by ANOVA analysis in ERK1/2 phosphorylation (pERK1/2). As expected, a significant increase of pERK1/2 (p < 0.05) was found in spinal cords of contusioned WT mice compared to WT naïve and WT sham. However, no significant differences (p > 0.05) were found between σ_1_R KO experimental groups, indicating that activation/phosphorylation of ERK did not significantly occur in lesioned σ_1_R KO mice (Fig. 3A). Accordingly, the pERK1/2 increase relative to corresponding naïves was significantly higher (p values < 0.05, Duncan test) in contusioned WT mice compared to both sham and contusioned σ_1_R KO mice. No significant changes in total ERK protein in both genotypes were found when the SCI groups were compared with their respective naïve or sham-operated groups.

**Figure 4:**
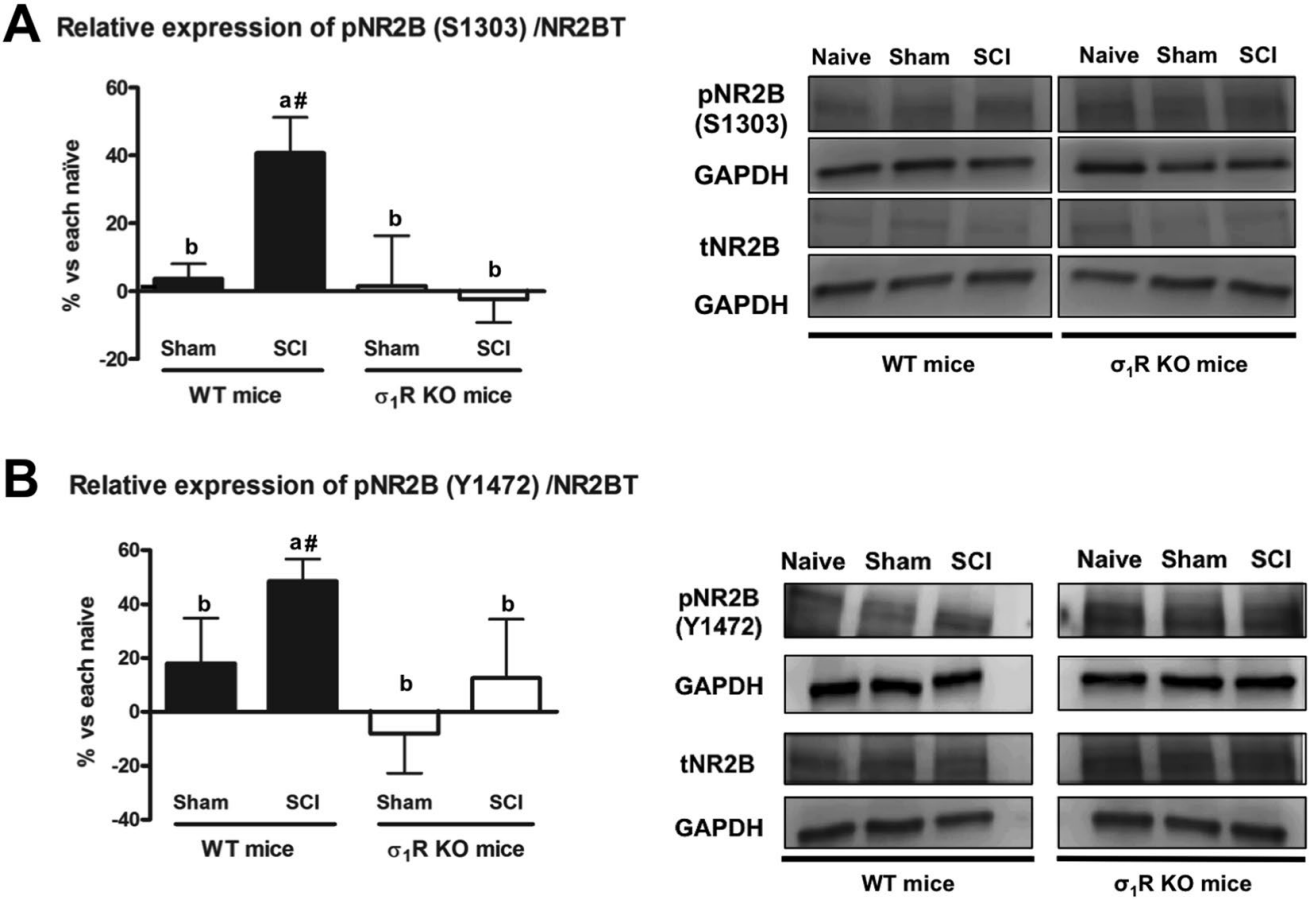
Spinal Tyr1472 and Ser1303 phosphorylation of N-methyl-D-aspartate (NMDA) receptor NR2B subunit at day 28 after spinal cord injury (SCI) in wild type (WT) and sigma-1 receptor (σ1R) knockout (KO) mice. (**A**) Quantification and representative immunoblots of total NR2B, pS1303NR2B and glyceraldehyde 3-phosphate dehydrogenase (GAPDH). (**B**) Quantification and representative immunoblots of total NR2B, pY1472NR2B and GAPDH. a–b: groups not sharing a letter are significantly different, p < 0.05; ^#^significant differences vs. naïve (p < 0.05). Results were presented as the mean ± standard error of the mean (n = 5–6). Protein expressions were normalized to GAPDH. Data was presented as a percentage respect to WT naïve or KO naïve mice. After SCI, increased levels of Tyr1472 and Ser1303 phosphorylation of NMDA receptor NR2B subunit were evidenced in WT mice, whereas no changes were detected in KO mice. Full-length blots are presented in Supplementary Figure S2.

Similarly, ANOVA analysis revealed significant group differences in Ser1303 and Tyr1472 phosphorylation of NMDA receptor NR2B subunit (p < 0.05). WT mice subjected to SCI (but not sham-operated animals) showed a significant increase in phosphorylation at Ser1303 in the NR2B subunit compared with the WT naïve group. In contrast to WT mice, levels of Ser1303 phosphorylation in NR2B were not significantly modified in σ_1_R KO mice subjected to either SCI or sham operation (Fig. 4A). Likewise, Tyr1472 phosphorylation in the NR2B subunit was significantly increased in SCI WT animals (p < 0.05) when compared with WT naïve animals, whereas no changes were found following SCI or sham operation in σ_1_R KO mice (Fig. 4B). No significant changes in the total levels of NMDAR-NR2B were observed in any of the experimental groups.

Overall, data point to prevention of pERK1/2 and pNR2B upregulation in the spinal cord as a molecular, mechanistic readout for the attenuation of SCI-induced pain-related behaviours in σ_1_R KO mice.

### Pro-inflammatory cytokines TNF-α and IL-1β are not upregulated in σ_1_R KO mice after SCI

**Figure 5:**
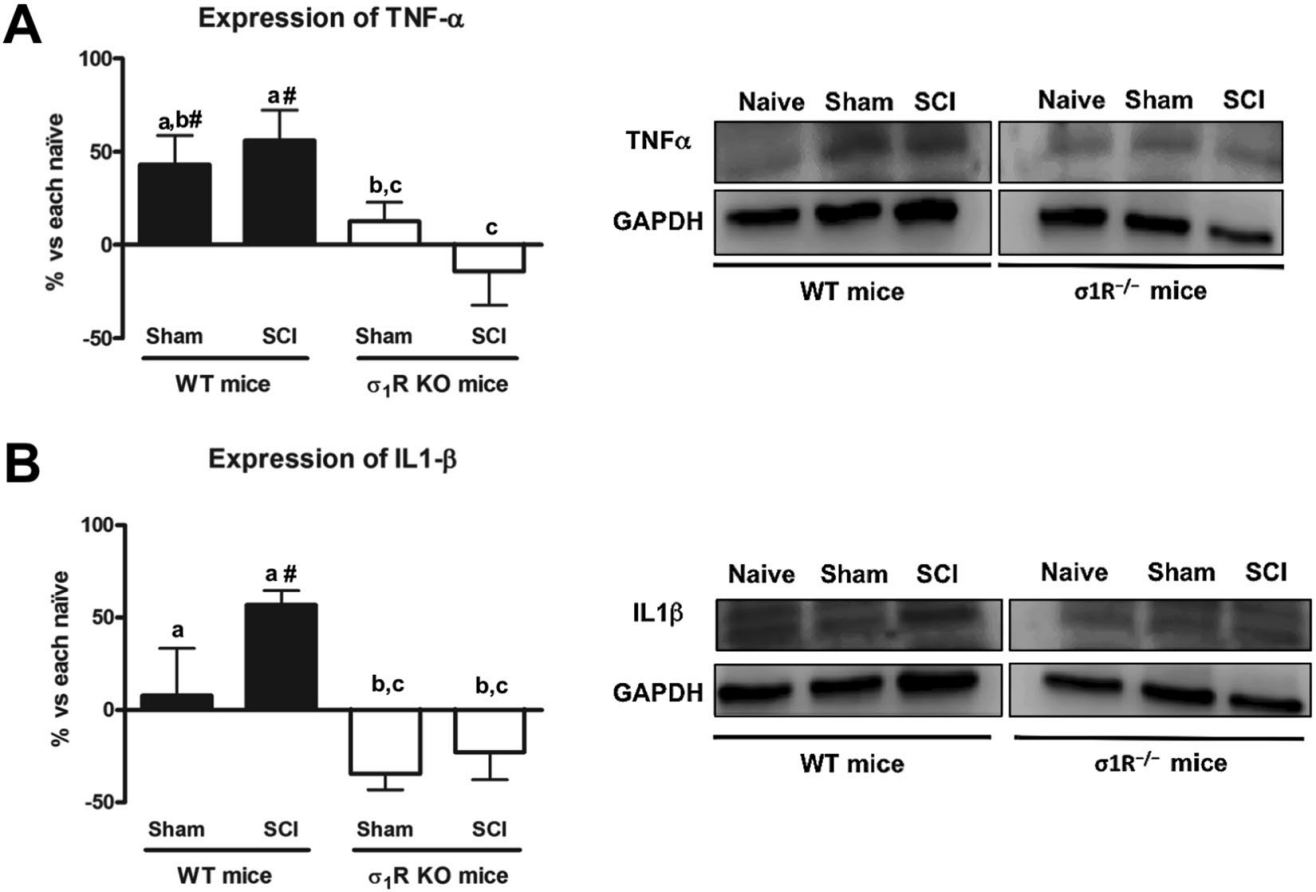
Spinal inflammatory cytokines (tumour necrosis factor [TNF]-α and interleukin[IL]-1β) expression at day 28 after spinal cord injury (SCI) in wild type (WT) and sigma-1 receptor knockout (KO). (**A**) Quantification and representative immunoblots of TNF-α and glyceraldehyde 3-phosphate dehydrogenase (GAPDH). a–b: groups not sharing a letter are significantly different, p < 0.05. (**B**) Quantification and representative immunoblots of IL-1β and GAPDH. a-c: groups not sharing a letter are significantly different, p < 0.05; ^#^significant differences vs. naïve (p < 0.05). Results were presented as the mean ± standard error of the mean (n = 5–6). Protein expressions were normalized to GAPDH. Data was presented as a percentage respect to WT naïve or KO naïve mice. After SCI, an increase in TNF-α and IL-1β protein levels in spinal cord was evidenced, whereas the deficiency of σ1R prevented its upregulation. Full-length blots are presented in Supplementary Figure S3.

Since the pro-inflammatory cytokines TNF-α and IL-1β have been associated with central neuropathic pain after spinal cord injury^33–35^, we also assessed whether their expression in spinal cord is attenuated in σ_1_R KO mice 28 days after injury. The ANOVA analysis revealed significant differences between groups in both TNF-α and IL-1β expression (p < 0.05). While contusioned WT mice (and WT sham-operated mice in the case of TNF-α) showed a significant upregulation of TNF-α (p < 0.05) (Fig. 5A) and IL-1β (Fig. 5B) in comparison to WT naïve animals, no differences (p > 0.05) were found between σ_1_R KO mice experimental groups, indicating that the expression of these pro-inflammatory cytokines in the spinal cord was not upregulated at 28 dpi in σ_1_R KO mice, subjected to either SCI or to sham operation.

### Acute σ_1_R receptor antagonist (MR309) administration reverses mechanical allodynia and thermal hyperalgesia in WT mice at day 28 after SCI

Data coming from previous experiments using genetically modified mice suggest that σ_1_R plays a role in central neuropathic pain development after spinal cord contusion and that the absence of this protein in KO mice results in attenuated neuropathic pain. In order to discard interfering adaptive changes in gene expression in the KO mice we investigated the effect of treatment with the selective σ_1_R antagonist MR309 (S1RA)^13^ on the nociceptive behaviour of spinal cord-injured WT mice. That is, does the absence of the protein in KO mice mimic the modulatory effect of pharmacologic antagonism when the protein is present in WT mice? To this end, we administered MR309, at doses that do not interfere motor coordination^13^, in an acute dose–response study (10, 20, 40 and 60 mg/kg) to WT mice at 28 dpi, and mechanical allodynia and thermal hyperalgesia were recorded 30 min after i.p. administration.

**Figure 6:**
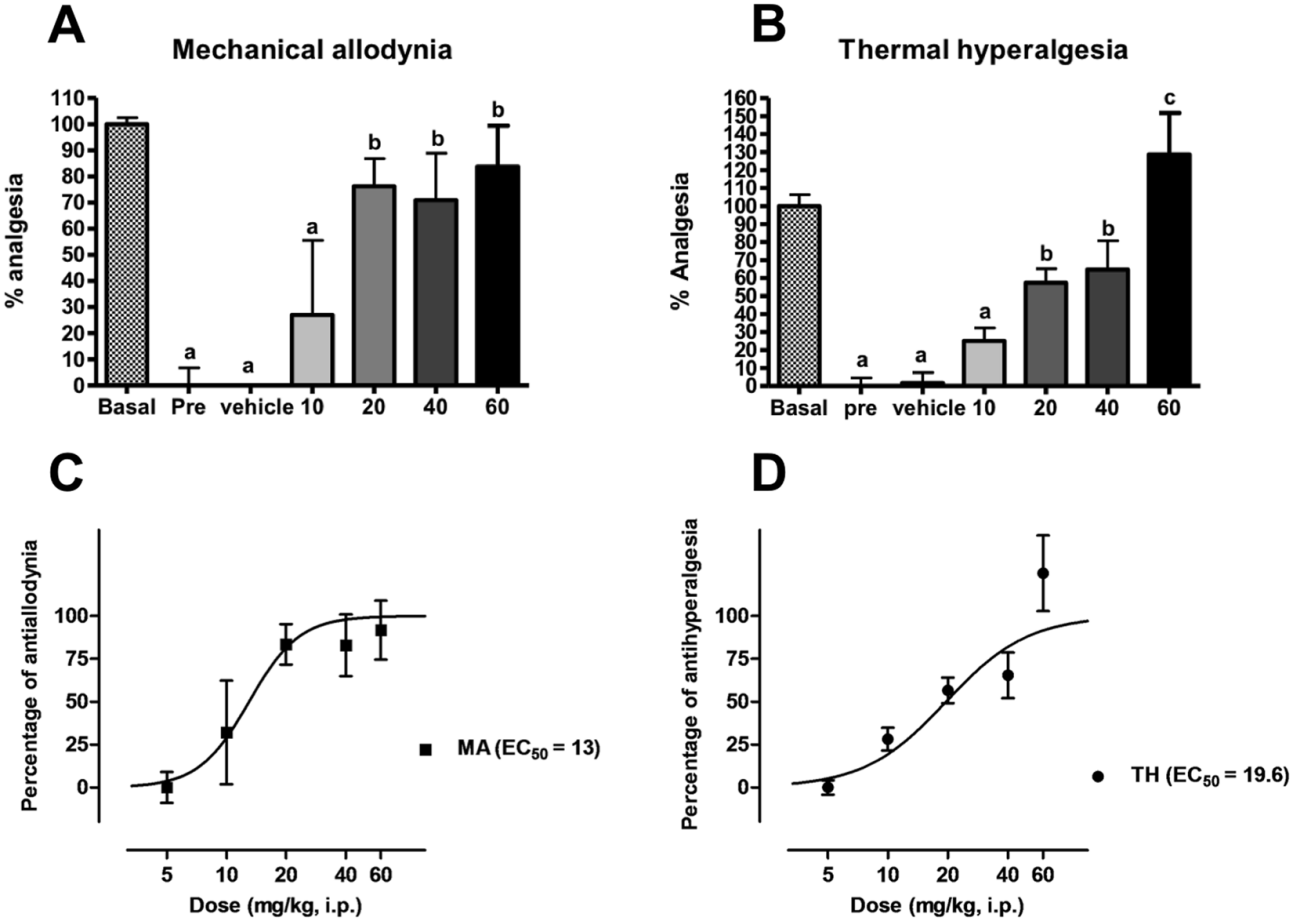
Dose–response effect of MR309 treatment on spinal cord injury (SCI)-induced mechanical allodynia and thermal hyperalgesia in wild type (WT) mice. Analgesic effects on (**A**,**C**) mechanical allodynia and (**B**,**D**) thermal hyperalgesia of the sigma-1 receptor antagonist MR309 (S1RA) 30 min after administration at 28 days after SCI. Each bar and vertical line represents the mean ± standard error of the mean (n = 4–7 per group). a–c: groups not sharing a letter are significantly different, p < 0.05. MR309 treatment dose-dependently reversed both mechanical and thermal hypersensitivity.

The administration of the σ_1_R antagonist elicited significant effects on mechanical allodynia for time-point (basal [i.e. pre-injury], pre-treatment and 30 min after treatment) (F_(2,25)_ = 135.055, p < 0.001) and dose (F_(4,26)_ = 2.642, p < 0.05) factors, and significant interaction for time-point × dose (F_(2,25)_ = 4.971, p < 0.001). Likewise, the MANOVA analysis of thermal hyperalgesia indicated significant effects for time-point (F_(2,25)_ = 98.154, p < 0.001) and dose (F_(4,26)_ = 4.394, p < 0.005) factors and significant interaction for time-point × dose (F_(2,25)_ = 5.566, p < 0.001). Thus, the administration of MR309 reversed both mechanical allodynia (Fig. 6A) and thermal hyperalgesia (Fig. 6B) in SCI WT mice with similar ED_50s_ (mechanical allodynia ED_50_ = 13 ± 3.14 mg/kg, Fig. 6C; and thermal hyperalgesia ED_50_ = 19.6 ± 2.54 mg/kg, Fig. 6D). ANOVA analysis of mechanical allodynia revealed that doses from 20 to 60 mg/kg (but not 10 mg/kg) exerted a similar and significant antiallodynic effect (p < 0.05) when compared to vehicle treatment. In the case of thermal hyperalgesia, the antinociceptive effect was significant from 20 to 60 mg/kg (p values < 0.05; but not at 10 mg/kg, p values > 0.05, Duncan test) and maximal at 60 mg/kg. Altogether, pharmacological results reinforce the findings in σ_1_R KO mice, without any apparent side effect observed.

## Discussion

In this study we used σ_1_R KO mice to determine the role of σ_1_R in central neuropathic pain-related behaviours triggered by spinal cord contusion. Our findings indicate that mechanical allodynia and thermal hyperalgesia found after SCI in WT mice are significantly attenuated in mice lacking σ_1_R, up to four weeks after injury. Moreover, our results indicate that attenuation of neuropathic pain-related behaviours in σ_1_R KO animals occurs concomitantly with a substantial reduction in the spinal cord of both ERK1/2 and NMDA-NR2B phosphorylation as well as with a reduced spinal expression of the pro-inflammatory cytokines TNF-α and IL-1β.

Normal sensory mechanical and thermal thresholds were not modified in naïve σ_1_R KO compared with WT mice, suggesting that σ_1_R KO perceive mechanical and thermal stimuli normally in the absence of lesion. However, when the nervous system becomes injured following SCI, nociceptive behaviours in response to mechanical and thermal stimuli were attenuated in σ_1_R KO versus WT mice. Similar results were obtained in previous studies where pain was induced at the periphery, with normal perception at baseline but increased nociceptive thresholds/latencies in σ_1_R KO mice following nerve injury^12,28^ or paw injection of chemical irritants^24–27^. In this study, mechanical allodynia was reduced an average of 54% and thermal hyperalgesia was reduced an average of 51% in σ_1_R KO versus WT mice. This agrees with findings supporting that σ_1_R plays a major role in the mechanisms underlying sensitization/hypersensitivity of pain pathways^14^. Accordingly, the spinal wind-up/amplification response to sustained stimulation of C- fibres is attenuated in isolated spinal cords from σ_1_R KO respect to WT mice^12^. It is also worth to mention that attenuated pain-related behaviours in σ_1_R KO were accompanied by slight but significantly lower BMS scores compared to WT mice. For the purpose of this study, not aimed at studying motor dysfunction after SCI, since differences are mild and BMS scores exceeded 6 points at day 28 in both genotypes, it may be stated that no major locomotor dysfunction occurs^40^ interfering the assessment and comparison of nociceptive readouts.

Observations in σ_1_R-deficient mice are supported by a pharmacological approach. The antiallodynic and antihyperalgesic effects exerted by the σ_1_R antagonist MR3069 on WT SCI mice here is in agreement with the antinociceptive effects of MR309 reported in a multitude of other models involving sensitization of pain pathways, such as formalin and capsaicin sensitization^13^, peripheral somatic or cephalic nerve injury^14,42^, chemotherapy-induced neuropathy^28,42^, diabetic-induced neuropathy^42^ or inflammation^26,27^. Behavioural findings (in animal pain models), as well as electrophysiological evaluations (spinal wind-up recordings) and neurochemical studies (spinal release of neurotransmitters) levels, provide evidence to suggest that σ_1_R antagonists inhibits hyperexcitability in sensitizing conditions: MR309 reduced the wind-up/amplification response to sustained stimulation of C-fibres in isolated WT spinal cords^13,15^ and inhibited formalin-evoked glutamate but enhanced noradrenaline release in the dorsal horn of the spinal cord^47^. This is in agreement with a modulatory effect on activity-dependent plastic changes, as a result of stimulating inhibitory pathways and dampening plastic excitatory changes in the dorsal horn. It thus seems that σ_1_R plays a major role in the mechanisms underlying activity-dependent plasticity/sensitization and ultimately pain hypersensitivity, perhaps irrespective of the primary lesion site and aetiology, and that the absence/blockade of σ_1_R inhibits such sensitization-related phenomena^14,48^.

We thus focused on some key central sensitization-related changes to reveal potential molecular pathways (either causative or consequential). We analysed the expression and activation (phosphorylation) of ERK1/2 which are known, together with other protein kinases, to be involved in central/spinal sensitization. ERK, a mitogen-activated protein kinase (MAPK), mediates intracellular signal transduction as a result of activation by a range of different stimuli. Phosphorylation of ERK in the dorsal horn in nociceptive neurons^49–52^ and/or reactive astrocytes^51–55^ has been described to play a major role (based on studies with ERK inhibitors) in hypersensitivity after peripheral nerve injuries. Spinal pERK levels can be increased due to traumatic injuries, particularly spinal injuries such as contusion, excitotoxic injury or chronic complete transection^56,57^. Our results in WT mice subjected to SCI agree with previous literature. Interestingly, in σ_1_R KO mice, spinal cord pERK1/2 remains unchanged following SCI, which would agree with the reduced mechanical and thermal hypersensitivity in these mice lacking σ_1_R. Previous studies also reported no changes in spinal pERK1/2 expression in peripheral nerve-induced or inflammatory pain models in σ_1_R KO mice: phosphorylation of ERK was increased in the ipsilateral spinal hemicord of WT mice but not in σ_1_R KO mice after partial sciatic nerve ligation^12^; and paclitaxel treatment induced peripheral neuropathy associated with pERK increase in WT but not in σ_1_R KO mice^28^. These findings suggest that σ_1_R facilitates ERK activation in the spinal cord in peripheral neuropathic pain animal models. In the present study, we provide evidence of a similar pattern in a spinal cord contusion model of central neuropathic pain, with complexities far beyond pain including outstanding neurodegenerative processes.

Activation of spinal dorsal horn NMDA receptors is essential for the initiation of central sensitization. NMDA receptors are formed by several subunits, with the NMDA-NR2B subunits reported to contribute to the development of central sensitization and neuropathic pain^58^. Specifically, it has been reported that NR2B phosphorylation of dorsal horn neurons is enhanced in neuropathic^59^ and post-inflammatory^60^ pain models, and the use of NR2B antagonists has been shown to reduce inflammatory^61,62^, peripheral^61,63^ and spinal cord injury-induced neuropathic pain^64^. Furthermore, σ_1_R activation has been associated with NMDA-NR2 phosphorylation in the hippocampus^65^ and this phenomenon was reduced in σ_1_R KO mice^23^. Glutamate is a key neurotransmitter and the NMDA receptor is a key channel for synaptic plasticity, mediates nociceptive transmission from afferent nociceptive fibres to dorsal horn spinal cord neurons, and promotes calcium entry and subsequent intracellular calcium-dependent signalling in dorsal horn neurons. Phosphorylation of NMDA receptor subunits mainly results in increased postsynaptic glutamate signalling (increased calcium permeability) and thus hyperexcitability/sensitization of spinal cord neurons. In particular, phosphorylation of NR2B at S1303, probably by protein kinase C (PKC), has been shown to potentiate NMDA receptor currents whereas phosphorylation at Y1472, probably by the protein tyrosine kinase Fyn, stabilizes active signalling NMDA receptors on the plasma membrane^66^. As expected, phosphorylation of NMDA-NR2B, presumably at PKC and Fyn sites, was increased in WT mice subjected to SCI compared to both naïve and sham WT animals. In contrast, phosphorylation of NMDA-NR2B at these sites (S1303 and Y1472) did not occur in σ_1_R KO mice following SCI. These findings suggest that σ_1_R facilitates NMDA receptor sensitization in the spinal cord in this SCI model of neuropathic pain. Accordingly, inhibition of NMDA receptor phosphorylation in mice lacking σ_1_R could contribute, at least in part, to the attenuated hypersensitivity of σ_1_R KO mice to mechanical and thermal stimuli after SCI. A role for σ_1_R in NMDA receptor sensitization in the dorsal horn is also supported by the enhancement by the σ_1_R agonists PRE-084 or carbetapentane and inhibition by the σ_1_R antagonist BD-1047 of the phosphorylation of NMDA-NR1 subunits at PKC and PKA sites in peripheral injury models^11,67^. At the molecular level, it has been reported that σ_1_R binds to NMDA-NR1 subunit and modulates NMDA receptors, and that σ_1_R antagonists (i.e., MR309) inhibit NMDA receptor function by dissociating σ_1_R from the NMDA receptor, thus allowing the interaction of the NMDA-NR1 receptor with calcium-calmodulin, a negative feedback regulator of NMDA function^22^.

Pro-inflammatory cytokines play a major role in neuron–glia cross-talk in neuroinflammatory processes but they also play a role in pain and central sensitization phenomena in the context of neuropathic pain^33–35,45,68–77^. In particular, spinal TNF-α and IL-1β are involved in mechanisms underlying the development and maintenance of neuropathic pain by promoting synaptic plasticity and central sensitization. In fact, intrathecal injection of TNF-α in naïve mice induces hind paw mechanical hypersensitivity, as observed in sciatic nerve-injured mice, and increased glutamatergic neurotransmission (a hallmark of spinal synaptic plasticity and sensitization), by decreasing astrocytic glutamate clearance from the synaptic cleft^78^. TNF-α also evokes a dramatic increase in the frequency of spontaneous excitatory postsynaptic currents (sEPSC) and NMDA receptor-mediated currents in lamina II neurons^79^. Accordingly, hyperalgesia elicited by intrathecal TNF-α is prevented by intrathecal injection of the NMDA receptor antagonist MK-801, thus providing further evidence of the role that TNF-α plays in regulating NMDA receptor-mediated synaptic plasticity and sensitization in the spinal cord^70,79^. A similar effect, leading to increased excitability of dorsal horn spinal neurons and facilitation of nociceptive transmission, is reported for IL-1β, including increased sEPSCs and decreased spontaneous inhibitory postsynaptic currents (sIPSC)^80^. In this study, the expression of both TNF-α and IL-1β was increased in WT but not in σ_1_R KO mice subjected to SCI 28 dpi, thus suggesting that inhibition of pro-inflammatory cytokine expression in mice lacking σ_1_R may contribute, at least in part, to the attenuated hypersensitivity to mechanical and thermal stimuli after SCI. Altogether, mechanistic correlates, including expression of extracellular mediators (i.e., TNF-α and IL-1β), signalling through excitatory membrane receptors/channels (i.e., NMDA receptors) and intracellular signalling cascades (i.e., ERK/pERK), converge to central sensitization-related phenomena as underlying substrates for the σ_1_R-mediated modulatory effect, which is consistent with previous literature supporting a central inhibitory effect of σ_1_R antagonism on other pain conditions involving spinal plasticity/sensitization and ultimately pain hypersensitivity^12–14^. Neuropathic pain is known to be induced when the mechanisms mentioned above are engaged in the spinal cord, and inhibition of the mechanisms mentioned above has been found to reduce neuropathic pain. However, it is unclear if these changes are causal in nature or just a consequence of changes upstream. Protection against excitotoxic damage via inhibition of glutamatergic NMDA signalling, as expected by σ_1_R antagonism based on the reported σ_1_R-NMDAR interaction^21^, could result in reduced neuronal stress and degeneration, and thus in reduced activation of microglia and astrocytes and reduced release of neuroinflammatory mediators. However, we don’t know if neuroprotection actually occurs and if it is upstream and plays thus a causative role. In fact, neuroprotection could also be a consequence of reduced glial activation and secretion of neuroinflammatory mediators.

σ_1_R expression was found to be increased in dorsal horn astrocytes following thoracic spinal cord hemisection, and administration of the σ_1_R antagonist BD-1047 on postoperative days 0–7 was found to inhibit expression of the astrocytic gap junction protein connexin 43, astrocyte activation in the lumbar dorsal horn, and ultimately mechanical allodynia^81^. These findings regarding allodynia modulation, although in a different model (hemisection *versus* traumatic contusion injury), further support a role for σ_1_R in central neuropathic pain following spinal cord injury. In the present work we used the contusion model as most of human injuries involve tissue damage due to impact against the spinal cord. Indeed, more than 500,000 people suffer acute traumatic SCI worldwide every year, and global prevalence is expected to increase^82^. We focused on three central sensitization-related mechanistic correlates, including extracellular mediators (i.e., TNF-α and IL-1β), membrane receptors/channels (i.e., NMDA receptors) and intracellular signalling cascades (i.e., ERK/pERK), and we first describe that increased spinal activity/expression of ERK, NMDA-NR2 and TNF-α and IL-1β after SCI did not occur in mice lacking σ_1_Rs, concomitant with attenuation of nociceptive behaviours. Functional activity/expression of all three central sensitization markers may require and/or engage astrocytes and perhaps microglia as well, and ultimately affect neuronal activity. It is clear that their functional activity/expression connects with synaptic plasticity, central sensitization and neuropathic pain. It is unclear, however, how σ_1_R modulates complex changes accounting for central sensitization in neuropathic pain and other pain sensitizing conditions, not only after peripheral injuries but also, specially, when the primary lesion occurs in the CNS.

## Methods

### Animals

Wild-type (WT) adult female CD1 mice that weighted a median of 22gr (19–26 gr) were obtained from Charles River Laboratories (France). In parallel, homozygous σ_1_R knockout (σ_1_R^*−/−*^; σ_1_R KO) mice with a median weight of 23gr (20–26 gr) were generated and characterized as described previously^83^, backcrossed onto the CD-1 background with selection for the mutant σ_1_R gene at each generation. After 10 generations of successive backcrosses with pure CD-1, mice harbouring the mutation were then bred to homozygosity and used in this study. Both WT and KO mice were 5-week–old (4–5 weeks old).

Mice were housed in a colony room at 21 ± 1 °C and 40–60% humidity, with a 12:12 hours light/dark cycle and access to food and water *ad libitum*, in groups of five in 331 × 159 × 132 cm plexiglass cages with a wood-shaving bedding. Cages were changed twice weekly. Behavioural testing was carried out in a soundproof experimental room. All mice were allowed to acclimatise to the facility rooms before commencing any behavioural or surgical procedures, which were all conducted during the light cycle. Sentinel mice were routinely tested for pathogens and facilities remained pathogen free during the whole experimental period.

All experimental procedures and animal husbandry were conducted following the ARRIVE guidelines and according to the ethical principles of the I.A.S.P. for the evaluation of pain in conscious animals^84^ and the European Parliament and the Council Directive of 22 September 2010 (2010/63/EU), and were approved by the Animal Ethics Committee of the Parc Científic of Barcelona.

### Experimental design and dosing

Two independent sets of experiments were conducted. Experiments in Part 1 were designed to study whether the lack σ_1_R may result in attenuation of pain-related behaviours in mice after central nervous system injury. To this end, CD-1 WT and σ1R KO female mice were subjected to SCI and their functional responses were assessed at 7, 14 and 28 dpi, including locomotor and nociceptive behaviours (mechanical allodynia and thermal hyperalgesia), comparing them with naïve and sham animals. Locomotor recovery was assessed first, using BMS scale in an open field maze, afterwards mechanical allodynia was measured by the Von Frey filament tests and finally, the thermal hyperalgesia was assessed by the plantar test. The behavioural tests were performed in this specific order and with an interval of 20 min between each test. Moreover, biochemical assays were performed to relate mechanical allodynia and thermal hyperalgesia findings to neuropathic pain biomarkers. All of these procedures are described below.

A second set of experiments (Part 2) was designed once results suggested significant attenuation of pain-related behaviours in σ_1_R KO mice after SCI. The objective of these experiments was to elucidate whether the σ_1_R antagonist MR309 (S1RA) decreased mechanical allodynia and thermal hyperalgesia in SCI WT mice. Dose–response studies (0, 20, 40, and 60 mg/kg i.p.; vehicle: hydroxypropyl methylcellulose) were performed at 28 days post-injury evaluating mechanical allodynia and thermal hyperalgesia 30 min after dosing, obtaining afterwards the percentage of analgesia.

A set of 26 WT and 30 σ_1_R KO mice was used to compare behavioural endpoints and molecular markers in both genotypes after spinal cord contusion, and another set of 31 WT mice was used to assess the effect of σ_1_R antagonist (MR309) administration on behavioural responses. Animal sample size was calculated using GRANMO (Version 7.12 April 2012).

### Surgical procedure

In order to obtain a mouse model of central neuropathic pain without locomotor paralysis, spinal cord contusion was conducted according to procedures explained elsewhere^35,41^. Briefly, animals were anesthetized with sodium pentobarbital (50 mg/kg, i.p.) and placed prone on a heating pad to maintain constant levels of body temperature. After back disinfection with povidone iodide, T8–T9 of the thoracic spinal cord was exposed via dorsal laminectomy, a metallic stage positioned over the exposed spinal cord and a 2 g weight then dropped onto the stage from a 25 mm height. Following this procedure, the wound was closed and animals allowed to recover in warmed cages with accessible food and water. After the surgical procedure, animals also received 0.5 mL saline solution (i.p.) to restore an eventual volemic deficit. In sham animals, the spinal cord was exposed as described above but not contusioned, and they underwent the same recovery procedures. Naïve mice did not receive any surgical manipulation. Mice were randomly allocated to experimental groups prior to surgical procedures.

### Locomotor activity

Locomotor activity was evaluated by means of the BMS test^40^ and performed as described elsewhere^35^. Briefly, animals were placed individually into an open field (72 cm × 72 cm) and allowed to move freely for 5 min. During this time the hind limb movements of the animal were scored for locomotor function according to the BMS^40^, which ranges from 0 (no hind limb movement) to 9 (normal movement and coordinated gait).

### Mechanical allodynia and thermal hyperalgesia tests

Mechanical allodynia was assessed via hind paw withdrawal from von Frey filament stimulation^43^. Mice were placed in test chambers with a metal mesh floor through which von Frey monofilaments (bending force range 0.04–2 g) were applied to the plantar surface. Paw withdrawal thresholds were measured using the up-down method paradigm. In this paradigm, the 0.4 g was applied first. The response to this filament determined which filament was applied next; a weaker filament was applied if the animal had responded to the previous filament, a stronger filament was applied if the animal did not respond to the previous filament. Clear paw withdrawal, shaking or licking were considered to be a response. This up-down procedure was limited to four assessments after the first response. Each filament was applied for 2 s, with inter-stimulus intervals of 5–10 s. Both hind paws were tested. The 50% paw withdrawal threshold was calculated using the Dixon formula: 50% paw withdrawal threshold (g) = [(10^(Xf + κδ)^/10 000)], where Xf is the value (in logarithmic units) of the final von Frey filament used, κ is a fixed tabular value for the pattern of positive/negative responses and δ is the mean difference (in log units) between stimuli. Both paws were evaluated since SCI model results in a bilateral injury and it is not possible to use contralateral paw as a natural intraindividual control.

Thermal hyperalgesia was assessed by determination of hind paw withdrawal latency in response to a thermal stimulus (radiant heat) administered via a plantar test analgesia meter (IITC, Life Science), according to the Hargreaves method^44^. Mice were placed into test enclosures, with the temperature-controlled (29 °C) glass surface of the plantar test device positioned directly underneath. Animals were then allowed to acclimatize for 45 min. The radiant heat source was then positioned under the plantar surface of the animal’s hind paw and activated. A light beam intensity that elicited baseline paw withdrawal latencies of 14–15 s was used. A maximum limit of 20 s was imposed in order to prevent tissue damage in the absence of a withdrawal response. The SCI model results in a bilateral injury, which does not allow the use of contralateral paw as a natural intraindividual control, so both paws were evaluated. The sum of the mean withdrawal latencies for both hindpaws were determined from the average of three separate trials, conducted at 5-min intervals.

### Biochemical assays

#### Genotyping of σ1R KO mice

Genotyping was conducted using genomic DNA obtained from tail tips of WT and σ_1_R KO mice. DNeasy Blood & Tissue kits (QIAGEN, Madrid, Spain) were used to analyse samples, according to the manufacturer’s instructions. PCR amplifications were conducted using Eppendorf Hot-MasterMix (Eppendorf, Hamburg, Germany) and 0.5 µM of each primer (Invitrogen Ltd, Paisley, UK). PCR was conducted using a thermal controller (iCycler, Bio-Rad Laboratories, Hercules, CA) with initial template denaturation at 94 °C, followed by 35 cycles of 30 s at 94 °C, 45 s at 55 °C and 2 min at 70 °C. As a final extension step, a10-min cycle was run at 72 °C. The oligonucleotide primer (5′–3′) sequences specific for the genes examined were: 5′-AAT TTT GCT CCC CTC CTC-30 and 50-CGT TCA CAA ATA CCC ACT G-3′ for the WT allele and 5′-GGA ACC AGA TGA CCC ACA GGT GC-30 and 50-CGC CAT TCA GGC TGC GCA ACT GTT GGG-3′ for the mutant allele^83^. A range of molecular weight markers (EZ Load Molecular Rulers, Bio-Rad Laboratories, Hercules, CA, USA) were also used. Amplified products were analysed by electrophoresis on 2% agarose gel containing ethidium bromide. Gels were photographed using an ultraviolet (UV) transilluminator to visualize stained bands. All animals used in the present study had the genotype corresponding to their experimental group.

#### Western blotting

Twenty-eight days after surgery, WT and σ_1_R KO mice (n = 4–6 per group) were euthanized with sodium pentobarbital (100 mg/kg, i.p.) and spinal cord T8–T9 segments immediately removed, frozen in dry ice and stored at −80 °C.

Spinal cord tissue was homogenized by sonication in TRIS buffer (50 mM Tris, 150 nM NaCl, 1% NP-40, 2 mM EDTA, 1 mM phenylmethylsulfonyl fluoride, Triton X-100, 0.1% SDS, 1mMNa3VO4, 25mMNa), 5% protease inhibitor cocktail and 1% phosphatase inhibitor cocktail (all from Sigma-Aldrich Quimica S.A). The resultant homogenate was then centrifuged at 10,000 G at 4 °C for 10 min. The supernatant was decanted from the pellet, and the protein concentration from the obtained supernatant was measured using a Lowry assay. Samples were then stored at −80 °C until use.

Thirty microgram samples were fractionated using 10% (w/v) SDS–PAGE and transferred onto a polyvinylidene difluoride membrane, blocked either with 5% non-fat dry milk or bovine serum albumin (BSA) in tris–tween 20-buffered saline (T–TBS) for 1 hour at room temperature. Membranes were then incubated with primary antibodies overnight at 4 °C: rabbit anti-extracellular signal-regulated kinases (total ERK 1/2) (1:40000, M5670, Sigma-Aldrich), diphosphorylated ERKs (pERK1/2)(1/:800, 44680 G, invitrogen) were diluted in T–TBS containing 1% non-fat dry milk. Rabbit anti-pY1472-GluN2B (1/:1000, M2442, Sigma-Aldrich), anti-pS1303-GluN2B (1/:3000, ab81271, Abcam), anti-GluN2B (1:750, ab15557P, Merk Millipore), anti-TNF-α (1/500, ab6671, Abcam) and anti-IL-1β (1/500, ab9722, Abcam) were also used and diluted in T–TBS containing 1% BSA solution. To ensure equal protein loading, rabbit anti-glyceraldehyde 3-phosphate dehydrogenase (GAPDH) antibody (1:40,000, Sigma-Aldrich Quimica S.A.) was used as a loading control. The blots were washed four times for 15 min with T–TBS and then incubated for 1 hour at room temperature with horseradish peroxidase–conjugated goat antirabbit IgG, purchased from Pierce Biotechnology Inc. (Rockford, IL, USA) and revealed by chemiluminescence (Immun-Star HRP Chemiluminescent Kit) from Bio-Rad. Chemiluminescence was detected with a C-DiGit^®^ Blot Scanner (Li-cor).

The densiometric analysis of immunoreactive bands was done using the Image Studio Lite 5.2 (LI-COR Bioscience). pERK,Y1472-GluN2B and pS1302-GluN2B were normalized to total ERK and total gluN2B respectively, and in turn, normalized with respect to the intensity of the corresponding GAPDH immunreactivity. TNFα and IL1β were also normalized to the corresponding GAPDH intensity.

### Statistical analysis

All functional and molecular biology measurements were performed in a blinded manner, using code numbers for both mice and samples. Results shown are mean ± SEM. Data were analysed using repeated measures MANOVA (Wilks’ criterion) and analysis of variance (ANOVA) followed by Duncan’s test, when applicable. In the pharmacological study, the percentage of antiallodynic or antihyperalgesic effect exerted by a treatment was calculated as follows: % effect = [(PWD − PWV)/(PWN − PWV)] × 100, where PWD and PWV are the paw withdrawal latency (s) or threshold (g) in drug-treated and pre-treated animals, respectively, and PWN is the paw withdrawal in naïve animals. A dose–response curve was plotted using nonlinear regression analysis, and the dose of drug that produced 50% of its maximal possible response (ED50) obtained. In all statistical analyses, the α level was set at 0.05. Statistical analyses were conducted using SPSS 23.0 for Windows.

### Data availability

All data generated or analysed during this study are included in this published article.

## Electronic supplementary material

Supplementary Information
