# Supplementary material for: Critical role of sigma-1 receptors in central neuropathic pain-related behaviours after mild spinal cord injury in mice
Source: Sci Rep. 2018 Mar 1;8:3873. doi: 10.1038/s41598-018-22217-9 (PMC5832850; doi:10.1038/s41598-018-22217-9)

## Supplementary Information

# Critical role of sigma-1 receptors in central neuropathic pain-related behaviours after mild spinal cord injury in mice

Sílvia Castany[1,2], Georgia Gris[2], José Miguel Vela[2], Enrique Verdú[1*], and Pere Boadas-Vaello[1*]

[1] Research Group of Clinical Anatomy, Embryology and Neuroscience (NEOMA), Department of Medical Sciences, Universitat de Girona (UdG), Girona, Spain.
[2] ESTEVE, Drug Discovery and Preclinical Development, Parc Científic de Barcelona, Barcelona, Catalonia, Spain.

**Supplementary Figure S1:** Full-length blot images corresponding to the cropped western blot presented in Figure 3

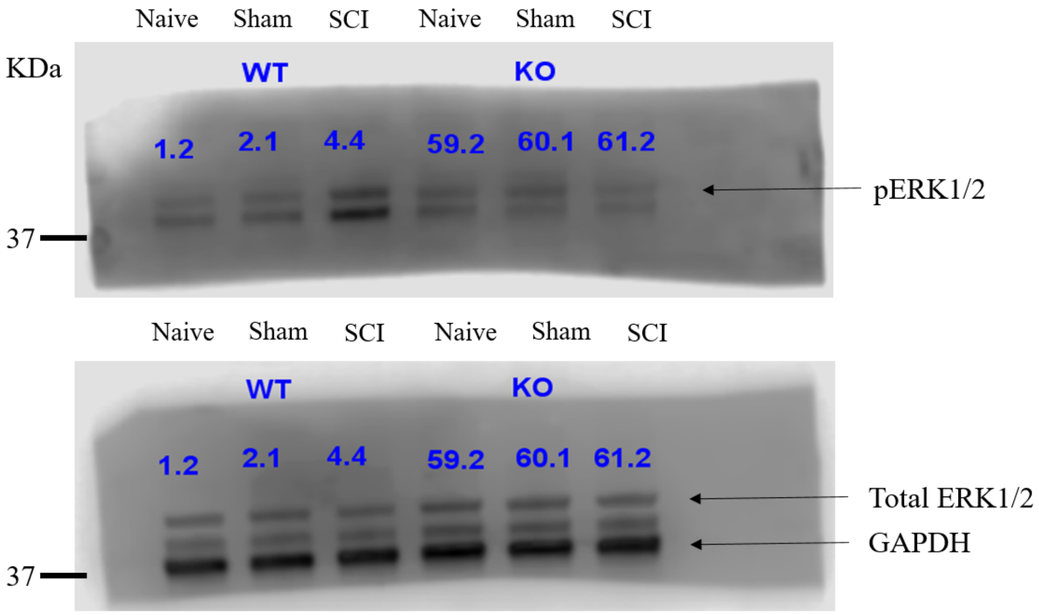

**Supplementary Figure S2:** Full-length blot images corresponding to the cropped western blot presented in (a) Figure 4A and (b) Figure 4B

**(a):**

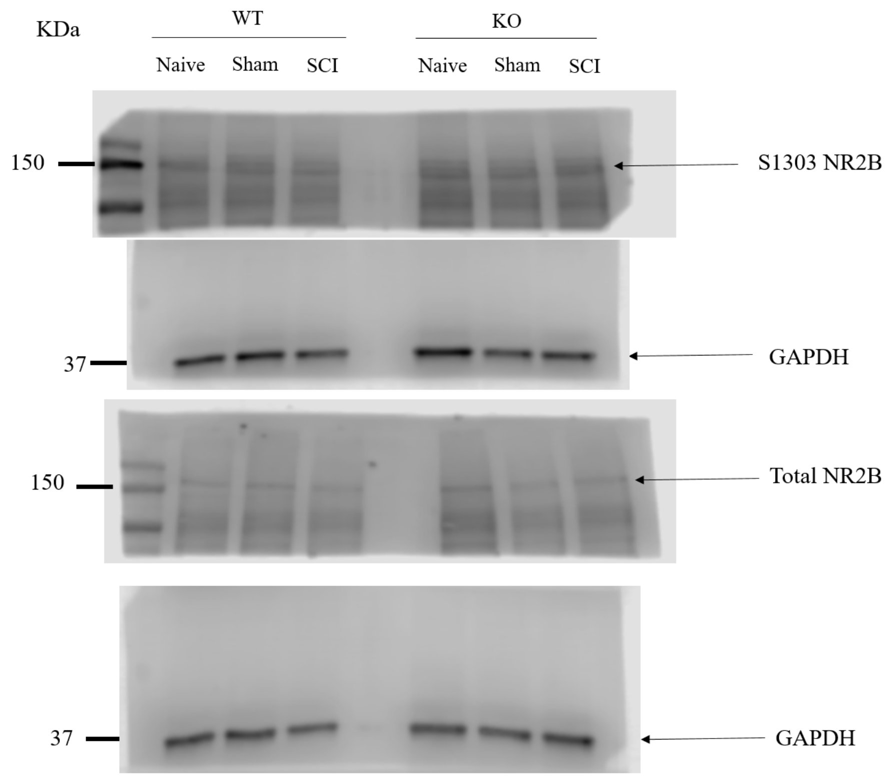


**(b):**

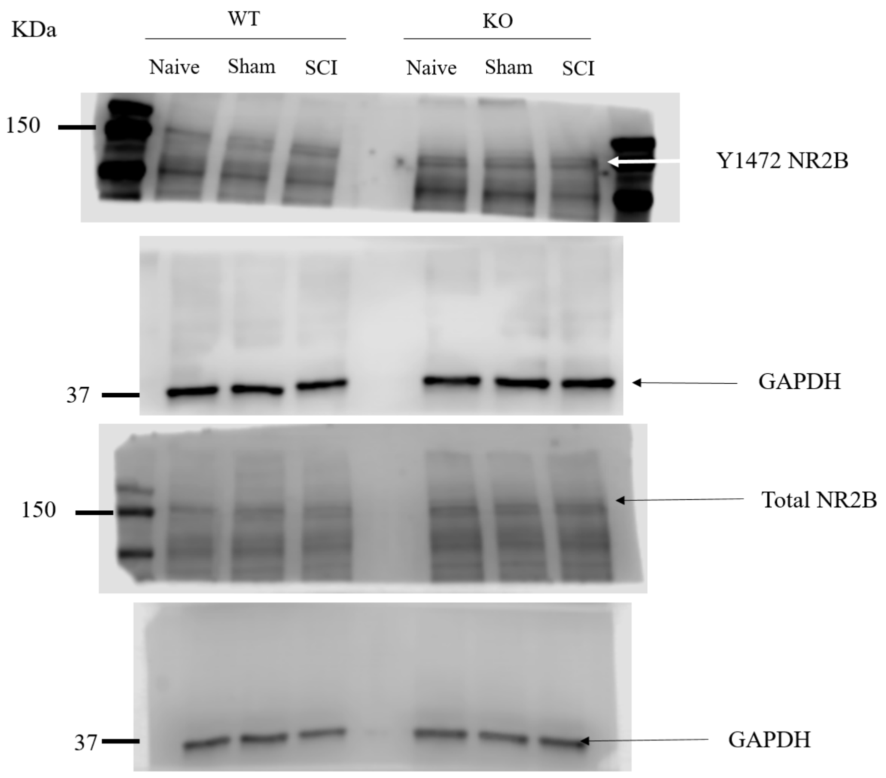

**Supplementary Figure S3:** Full-length blot images corresponding to the cropped western blot presented in (a) Figure 5A and (b) Figure 5B

**(a):**

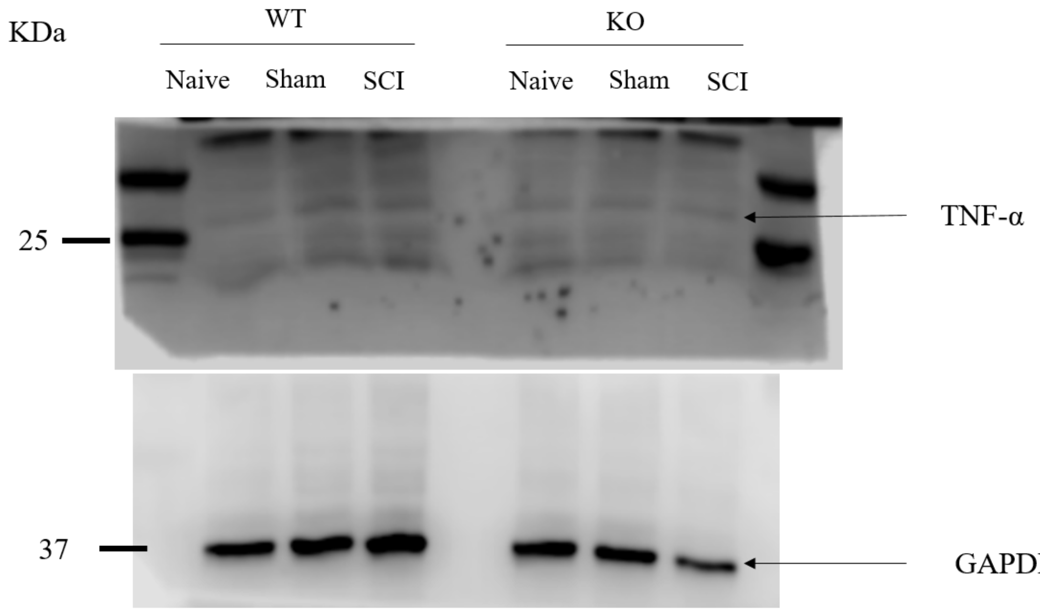


**(b):**

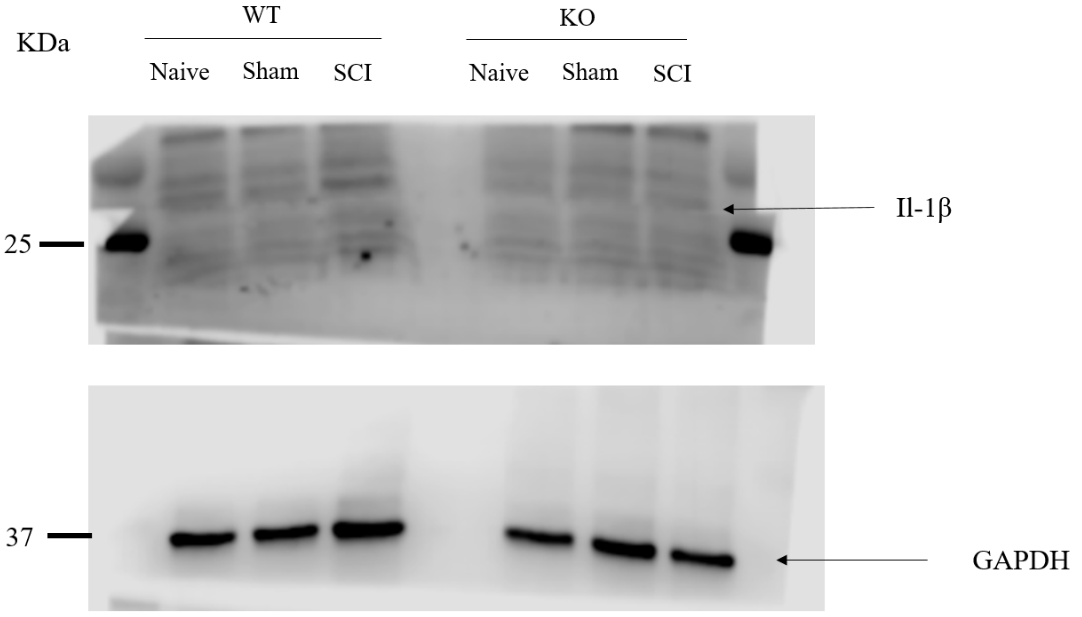

Supplement: Supplementary file 1 — Supplementary Information [file 41598_2018_22217_MOESM1_ESM.pdf]
